# First District Dental Society of New York

**Published:** 1892-03

**Authors:** 


### FIRST DISTRICT DENTAL SOCIETY OF NEW YORK.

THE annual meeting of this Society took place on the 18th, 19th, and 20th of January, and was carried through nearly according to announcement.

The attendance, while not equal to former occasions, was satisfactory, some two hundred from various sections of the country being present. These mainly composed the meeting, as the members of the dental profession from New York and vicinity were exceedingly limited in number. This fact created a good deal of comment and had a depressing effect upon those who took part. The weather was exceptionally bad, and contributed its share towards the feeling prevalent that this meeting was not equal to those which had preceded it in former years. Several of those who had accepted appointments failed, both in person and papers, to be present, but notwithstanding this the time was fully occupied by essays of excellent quality.

Whether this meeting is an indication of a growing feeling that local celebrations have had their day, or whether it means simply a temporary loss, it must be recognized that there are indications that it will be more and more difficult to secure the co-operation of the profession at large in holding these conventions with the enthusiasm that was formerly in unstinted measure accorded them. Indeed, we question whether they have not outlived the possibilities for usefulness, heretofore so freely conceded.

This, if it be true, is to be regretted, as they have certainly had a stimulating effect in the past very nearly equal to the more pretentious yearly gatherings, and have elicited the warm interest of persons widely separated, thus cementing bonds of professional friendship and advancing scientific interests.

